# Medical student resilience and stressful clinical events during clinical training

**DOI:** 10.1080/10872981.2017.1320187

**Published:** 2017-05-02

**Authors:** Jennifer C. Houpy, Wei Wei Lee, James N. Woodruff, Amber T. Pincavage

**Affiliations:** ^a^ Department of Medicine, University of Chicago, Chicago, IL, USA

**Keywords:** Burnout, resilience, wellness, clinical training, Undergraduate Medical Education

## Abstract

**Background**: Medical students face numerous stressors during their clinical years, including difficult clinical events. Fostering resilience is a promising way to mitigate negative effects of stressors, prevent burnout, and help students thrive after difficult experiences. However, little is known about medical student resilience.

**Objective**: To characterize medical student resilience and responses to difficult clinical events during clinical training.

**Design**: Sixty-two third-year (MS3) and 55 fourth-year (MS4) University of Chicago medical students completed surveys in 2016 assessing resilience (Connor Davidson Resilience Scale, CD-RISC 10), symptoms of burnout, need for resilience training, and responses to difficult clinical events.

**Results**: Medical student mean resilience was lower than in a general population sample. Resilience was higher in males, MS4s, those without burnout symptoms, and students who felt able to cope with difficult clinical events. When students experienced difficult events in the clinical setting, the majority identified poor team dynamics among the most stressful, and agreed their wellbeing was affected by difficult clinical events. A majority also would prefer to discuss these events with their team later that day. Students discussed events with peers more than with attendings or residents. Students comfortable discussing stress and burnout with peers had higher resilience. Most students believed resilience training would be helpful and most beneficial during MS3 year.

**Conclusions**: Clinical medical student resilience was lower than in the general population but higher in MS4s and students reporting no burnout. Students had some insight into their resilience and most thought resilience training would be helpful. Students discussed difficult clinical events most often with peers. More curricula promoting medical student resilience are needed.

## Background

Rates of burnout [1,2] and depression [1–5] are high among medical students. Burnout in medical students has been associated with self-reported unprofessional conduct and decreased altruism [6]. Additionally, students experiencing burnout during at least one measured time point in a longitudinal study were shown to have an increased likelihood of depression, a less positive perception of the learning environment, and more stress and fatigue [7]. Depression symptoms have been attributed to stress from the medical school environment more than from personal stressors alone [8]. While stress, burnout, and depression in medical students have been studied extensively, much less is known about medical student resilience, particularly in the United States.

Resilience is a measure of the ability to cope with stress and thrive when faced with adversity [9]. Fostering resilience is a promising way to mitigate the negative effects of stressors, prevent burnout, and help students succeed after difficult experiences. Higher levels of resilience have been associated with better subjective well-being in medical and nursing students in Finland [10], lower levels of distress in medical and psychology students in Australia [11], moderating negative life events in medical students in China [12], and higher quality of life scores and more positive perception of the educational environment in medical students in Brazil [13].

Resilience has been shown to be both quantifiable and modifiable [9,14], indicating it is a relevant point of study, and recently, it has been designated a priority area for medical education initiatives [15]. Canadian medical students have been shown to have lower resilience than age and gender matched counterparts in the general population [16]. This, combined with the statistics on medical student depression and burnout, indicates that medical student resilience warrants further characterization and intervention.

Because medical students encounter new, salient stressors during the clinical years, resilience could prove particularly helpful during this period. New stressors and challenges during the clinical years include patient death and dying [17–19], perceptions of unfair treatment, difficult team dynamics [19,20], and uncertainty [17,21] in both the clinical and learning environments. These challenges occur while students may be separated from their usual sources of peer support [19]. While medical student exposure to traumatic events during clinical rotations (as designated by ‘DSM-IV PTSD diagnostic criteria A1 and A2’ [22]) has been associated with personal growth, self-reported exposure to other stressful events during clinical rotations has been associated with higher depression and stress symptoms [22]. Perhaps as a result, the third year of medical school has been associated with a decline in empathy [23].

Understanding student reactions to difficult clinical events, especially reflection and discussion practices following these events, could help identify possible points of intervention for resilience training during the clinical years. The objective of this study was to perform an initial characterization of medical student resilience and responses to difficult clinical events.

## Methods

### Setting

We conducted a cross-sectional study at the University of Chicago Pritzker School of Medicine (PSOM). We provided an email link to an electronic survey to all 94 third year and 83 fourth year medical students. The third year curriculum at PSOM includes required clerkships at the University of Chicago Medical Center and its clinical affiliates in four 12-week blocks: Internal Medicine with Radiology (12 weeks); Surgery with Anesthesia (12 weeks); Pediatrics (6 weeks) and Obstetrics and Gynecology (6 weeks); Psychiatry (4 weeks), Family Medicine (4 weeks), and Neurology (4 weeks). Only Family Medicine and Neurology may be deferred to the fourth year. The fourth year clinical curriculum includes a required Emergency Medicine rotation (1 month) and a subinternship (1 month). The majority of students participate in additional clinical rotations.

Surveys were available for less than one month during the Spring of 2016, after Match Day. This time period encompassed the end of one and the beginning of another third year clerkship block. It also encompassed portions of two month-long fourth year blocks.

### Design

Participation was voluntary and anonymous. This study was granted an exemption by the Institutional Review Board at the University of Chicago. Surveys were conducted via Survey Monkey (www.surveymonkey.com).

Surveys assessed resilience, symptoms of burnout, need for resilience skills training, and responses to difficult clinical events. (For entire survey with proprietary CD-RISC 10 questions removed, please see Appendix).

### Measures

We assessed resilience using the 10-item version [24] of the Connor Davidson Resilience Scale [9] (CD-RISC 10). The scale asks respondents to rate how true (on a scale of 0 to 4) 10 statements are with respect to the respondent. Possible scores range from 0 to 40, with 40 representing a more resilient score.

We assessed symptoms of burnout using the validated [25] non-proprietary single-item burnout measure used in the Physician Work Life Study [25,26]. The responses are often dichotomized into ‘no symptoms of burnout’ (response of 1 or 2) or ‘one or more symptoms’ (response of 3, 4, or 5) [25,26]. We presented students with a list of clinical events and students indicated which events they had experienced during their clinical time. They also indicated which were the most stressful, when they would prefer to discuss them with their team, and with whom they had discussed them. If they indicated that they did not discuss difficult clinical events with attendings, they were asked to provide a reason. Students also used a 5-point Likert scale to indicate their agreement with several statements about their skills in dealing with difficult experiences and their need for additional resilience training. Finally, students selected the top (from a list) resilience skills to address in future workshops. We developed the lists of clinical events and topics for training based upon the authors’ experience (AP) moderating reflections sessions with clerkship students about difficult clinical events for 5 years and researching and teaching resilience skills to internal medicine residents for 2 years, as well as, the experience of the authors providing counseling for medical students across the continuum as deans of the medical school (JW & WL).

### Analysis

We compared descriptive statistics using chi-square tests and T-tests as appropriate. We used Stata version 13.0 software (StataCorp Lt).

## Results

### Resilience: demographics and symptoms of burnout

Sixty-two MS3s (response rate 62/94 = 66.0%) and 55 MS4s (response rate 55/83 = 66.3%) completed the survey. Demographic characteristics of the medical students surveyed are in Table 1. The mean CD-RISC 10 score was 28.21 (6.37) (range 10–40). (Results reported as mean (SD).) This was lower than in a general population sample (a community random digit dial sample of adults in Memphis who received the same CD-RISC 10 questionnaire) [27] (31.8 (5.4) n = 764 (71.5% female), p < 0.001) and a sample of Canadian medical students (29.74 (4.88) n = 149 (62.4% female), p = 0.039) [16].

**Table 1.** Medical student sample demographics, Pritzker school of medicine, 2016.

	x/N	%
*Sex*		
Female	68/117	58.1
Male	49/117	41.9
*Age*		
18–25	37/117	31.6
26+	80/117	68.4
*Undergraduate Major*		
Science	82/117	70.1
Non-science	35/117	29.9
*Year in School*		
MS3	62/117	53.0
MS4	55/117	47.0
*Path to Medical School*		
Traditional (straight from college)	55/117	47.0
Non-traditional (took time off)	62/117	53.0
*Symptoms of Burnout*		
No Symptoms of Burnout	69/114	60.5
1 or More Symptoms	45/114	39.5

Mean resilience was higher in males (30.47 (6.14) vs. 26.43 (6.02) p = 0.001) and MS4s (29.68 (5.98) vs. 26.91 (6.47), p = 0.02) (Table 2). There was no significant difference based on age, undergraduate major, or path to medical school. Resilience was also higher in students reporting no symptoms of burnout (30.44 (5.44) vs. 25(6.29), p < 0.001). (Table 2).

**Table 2.** Medical student resilience score by demographics and symptoms of burnout, Pritzker school of medicine, 2016.

	N	Sample MeanCD-RISC 10 (SD)	p
*Sex*			
Female	60	26.43 (6.02)	0.001
Male	47	30.47 (6.14)	
*Age*			
18–25	34	28.06 (6.10)	0.87
26+	73	28.27 (6.54)	
*Undergraduate Major*			
Science	75	28.96 (6.13)	0.07
Non-science	32	26.44 (6.67)	
*Year in School*			
MS3	57	26.91 (6.47)	0.02
MS4	50	29.68 (5.98)	
*Path to Medical School*			
Traditional (straight from college)	50	29.12 (5.68)	0.16
Non-traditional (took time off)	57	27.40 (6.88)	
*Symptoms of Burnout*			
No Symptoms of Burnout	63	30.44 (5.44)	<0.001
1 or More Symptoms	44	25 (6.29)	

10 item Connor Davidson Resilience Score (CD-RISC 10) by demographics and burnout.

### Difficult clinical events

**Figure 1.**
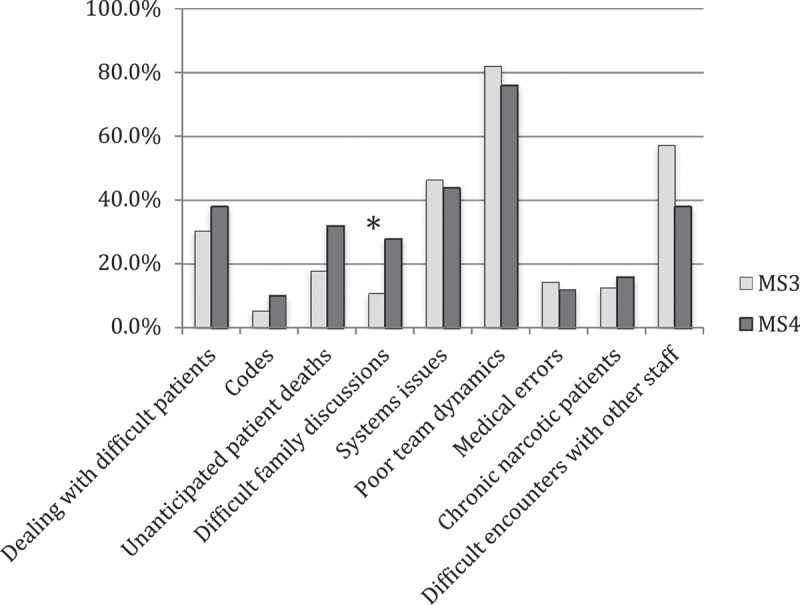
Clinical events identified as ‘most stressful’ by MS3 and MS4s. *p = 0.02 for comparison of MS3 vs. MS4 response. Students asked to identify three most stressful clinical events from a given list. MS3, third year medical students (n = 56); MS4, fourth year medical students (n = 50).

A large majority (over 80%) of students had experienced the following clinical events: dealing with difficult patients, difficult family discussions, systems issues, poor team dynamics, chronic narcotic patients, and difficult encounters with other staff. About half (54.7%, 58/106) had experienced medical errors. The four clinical events most often identified as among the three ‘most stressful’ were poor team dynamics (79.2%, 84/106), difficult encounters with other staff (48.1%, 51/106), systems issues (45.3%, 48/106), and dealing with difficult patients (34.0%, 36/106) (Figure 1). Exposure to these four events was not different among MS3s or MS4s. About half of students (58.7%, 61/104) agreed that difficult clinical events affect their wellbeing.

After difficult clinical events, the majority of students reflected on them often (70.5%, 74/105) and would prefer to discuss them with their team immediately (16.2%, 17/105) or later that same day (61.9%, 65/105). Only 4.8% (5/105) of students preferred not to discuss the events with their team at all.

**Figure 2.**
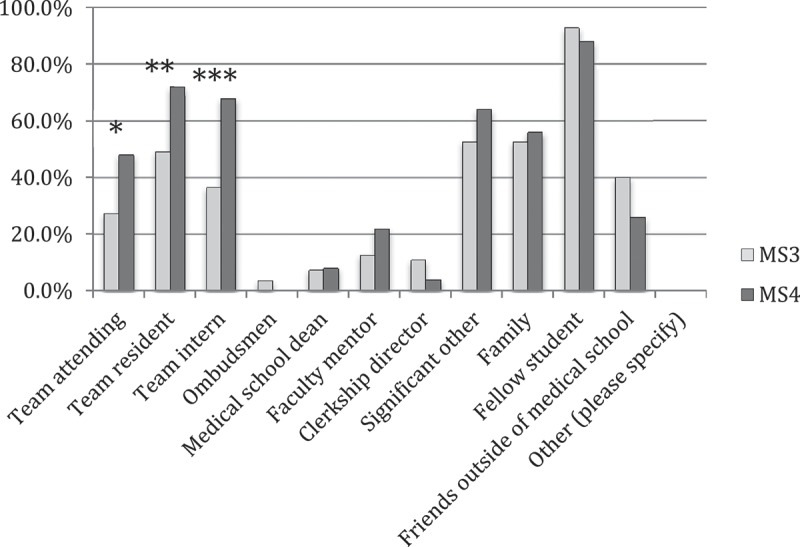
MS3 and MS4 percent responses to the question ‘Who have you talked to about difficult clinical events?’. *p = 0.043, **p = 0.017, ***p = 0.001 for comparison of MS3 vs MS4 response. MS3, third year medical students (n = 55); MS4, fourth year medical students (n = 50).

Most students (90.5%, 95/105) had talked to peers about difficult clinical events, while only 37.1% (39/105) had discussed them with the team attending and 60.0% (63/105) with the resident (Figure 2). More MS4s than MS3s discussed the events with attendings and residents (attendings: 48.0%, 24/50 vs. 27.3%, 15/55; p = 0.04; residents: 72.0%, 36/50 vs. 49.1%, 27/55; p = 0.02).

Students reported several reasons for not speaking to their attendings, including perceptions that their attendings were not receptive, available, or understanding. For example, one student commented that ‘The student’s interpretation of an event may be completely different than that of the attending’s, and as a result the student’s perspective may [not] be fully appreciated.’ Other students highlighted the limitations of their relationship with the attending, explaining that they were not close enough with their attending or didn’t feel that such a discussion would be appropriate given the team hierarchy or in their best interest given that the attending would grade them. For example, one student explained that ‘Often my stressors have to do with team dynamics, and I feel that (sadly) part of medical culture is that it is unacceptable to voice concerns about team dynamics to superiors.’

Students who reported they had the skills to cope with difficult clinical events (mean (SD) 29.47(5.91) vs. 22.89(5.93), p = < 0.001) and students who reported being comfortable discussing medical errors they were involved in with peers (30.48 (5.99) vs. 25.14 (5.64), p < 0.001) were more resilient. Likewise, students who reported being comfortable talking about stress and burnout with peers had higher resilience scores (29.36 (5.90) vs. 25.37 (6.91), p = 0.01).

### Resilience training

Most students (63.5%, 66/104) believed resilience training would be helpful, and only a minority (26.9%, 28/104) believed they had sufficient resilience training. Those that believed they had sufficient resilience training had higher resilience scores (mean (SD) 30.79 (6.77) vs. 27.42 (6.04), p = 0.03). Most students (65.7%, 67/102) believed resilience training would be most beneficial during the MS3 year. The topics most often identified as important for resilience training included coping with difficult team interactions (65.7%, 67/102), finding meaning in daily work (44.1%, 45/102), and dealing with disappointment/setbacks (43.1%, 44/102).

## Discussion

To our knowledge, this is the first study of resilience in US medical students that characterizes the relationships between several different demographics and resilience, as well as the first study that characterizes the relationship between resilience and self-perceptions of specific skills and symptoms of burnout. In our initial study, resilience in junior and senior medical students was lower than in the general population and a sample of Canadian medical students [9]. Male resilience scores were higher than female resilience scores, consistent with some [12,14], but not all [11] previous international findings. In addition, we identified resilience was higher in MS4s and students reporting no symptoms of burnout.

There are several possible explanations for our resilience findings. Since the Canadian study included all four years of medical school, it is possible that the resilience scores from the pre-clinical years increased the mean score. It is also possible cultural, demographic, or other training factors are responsible. MS4 resilience scores may be better because they have more control over their schedules, less rigorous schedules, and more clinical experience. Also, since MS4s were surveyed after Match day, their resilience and burnout scores may reflect a drop in stress and lighter schedule compared to the rest of the fourth year. Our finding that resilience in third and fourth year medical students was lower than in a general population sample is important and needs further investigation. Our findings also highlight that resilience of medical trainees is an important area for future study.

Students experienced many difficult clinical events and found poor team dynamics most stressful. This is likely due to their role in the hierarchy, stress of evaluation, and vulnerable position as a medical student. We believe students did not perceive medical errors or patient care events as stressful given their lack of autonomy and because they don’t yet feel responsible for patient care. Although the majority of students often reflect individually on difficult clinical events and want to discuss them with their team, most students discussed these clinical events with their peers rather than their team. Students reported that they rarely talk about these events with their attendings due to a variety of reasons, including limitations in perceived attending understanding, their role in the hierarchy, and concerns about grading. Interestingly, MS4s were more likely to speak to the team attending and resident which may be due to MS4s becoming more focused on their area of specialty, decreased emphasis on grades, and advancement of their role on the team. Further study on this area is needed.

Students who were comfortable speaking to their peers about stress and burnout or medical errors were more resilient. This is concordant with previous findings that an ‘approach-oriented’ rather than ‘avoidant-oriented’ strategy was associated with decreased burnout [1], and an ‘engagement’ rather than ‘disengagement’ strategy was associated with fewer depressive symptoms [5]. It may be that resilient students’ tendency to ‘bounce back’ allows them to speak more comfortably with their peers. Alternatively, a peer support system that encourages participants to discuss these events may help to build resilience. The latter possibility supports peer discussions and group reflection, which have been identified as important for resilience in previous articles [28–30], as possible targets for resilience training. Interestingly, students had some insight into their level of resilience, and this finding may be useful for future training. In concordance, students endorsed a need for further resilience training, preferably during the MS3 year.

There are some limitations to our study. Our study was conducted at a single medical school, and this could limit the external validity of the results. Due to non-response, selection bias may be present. The timing of the study included the transition between two clerkship blocks for the MS3s and between two month-long blocks for the MS4s. Responses may have been different had the study been conducted at a different time of year. Clinical event data was based on self-report, which may introduce bias. Additionally, because we asked students to reflect on their clinical experience as a whole when answering some questions, the answers may be subject to recall bias. However, if this is the case, answers likely represent memories that are most salient about clinical events, and thus may still provide useful, though slightly different, information. Additionally, some of the survey questions were not validated, thus students may have interpreted the question differently than intended. Furthermore, the data collected demonstrate several statistically significant associations but do not provide adequate information to outline causal relationships. Studies randomizing students to skills interventions versus standard training could help better elucidate the causal relationship between resilience traits and more favorable outcomes.

In summary, medical student resilience in clinical students was lower than in the general population and another medical student sample, but higher in MS4s and students reporting no symptoms of burnout. Students discussed difficult clinical events with peers more than with attendings or residents. Students had some insight into their level of resilience, and most thought resilience training would be helpful. More curricula promoting medical student resilience are needed, specifically focused on skills to help students cope with difficult team interactions. Additional, multi-institutional studies are needed to confirm our findings across the national population of medical students and look at more longitudinal variation. However, our findings can inform future study, suggest a need for further resilience training for clinical medical students, and guide development of educational interventions.

## Previous Presentations

An abstract containing a significant portion of the content of this article was presented at the 2016 Society of General Internal Medicine Midwest Regional Meeting, the 2017 Clerkship Directors of Internal Medicine Academic Internal Medicine week, and the 2017 Society of General Internal Medicine National Meeting.

## Appendix

Please do not put your name on this survey; we will be using your anonymous responses for research purposes. Your participation is voluntary.

**Sex**: Female Male **Age**: 18–2526+ **Undergraduate major**: Science Non-science

**Year in School**: MS3 MS4 **Path to medical school**: Traditional (straight from college) Non-traditional (took time off)

**Overall, based on your definition of burnout, How would you rate your level of burnout? (circle one):**

1. (1)‘I enjoy my work. I have no symptoms of burnout’
2. ”Occasionally I am under stress, and I don’t always have as much energy as I once did, but I don’t feel burned out”
3. ‘I am definitely burning out and have one or more symptoms of burnout, such as physical and emotional exhaustion’
4. ‘The symptoms of burnout that I’m experiencing won’t go away. I think about frustration at work a lot’
5. ‘I feel completely burned out and often wonder if I can go on. I am at the point where I may need some changes or may need to seek some sort of help’

**Connor Davidson Resilience scale (CD-RISC 10) was asked in its entirety**

**Which of the following clinical events have you experienced during your clinical time? (Mark all that apply)**

__Dealing with difficult patients__Codes__Unanticipated patient deaths__Difficult family discussions__Systems issues

__Poor team dynamics__ Medical errors__Chronic narcotic patients__Difficult encounters with other staff

**Which are the 3 most stressful clinical events for you? (Mark 3**):

__Dealing with difficult patients__Codes__Unanticipated patient deaths__Difficult family discussions__Systems issues

__Poor team dynamics__ Medical errors__Chronic narcotic patients__Difficult encounters with other staff

**After difficult clinical events, when would you prefer that your team discuss them? (circle one)**:

**Table:**

Immediately	Later the same day	In the following days to weeks	After the rotation is over	I would prefer not to discuss with my team

**After difficult clinical events, how often do you reflect on them individually? (circle one**):

Never Rarely Sometimes Often Very Often

**Who have you talked to about difficult clinical events? (Mark all that apply**):

_ team attending_team resident_team intern_ombudsmen_medical school dean_faculty mentor_clerkship director

_ significant other_family_fellow student_friends outside of medical school_other: ___________

**If you didn’t talk with your team attending, why not? (not required to respond) _______________________________________**

**_______________________________________________________________**

**Please state how strongly you agree or disagree with the following statements (mark one)**:

**Table:**

	Strongly Disagree	Disagree	Neutral	Agree	Strongly Agree
I have the skills necessary to personally cope with difficult clinical events (i.e. unexpected deaths, difficult patients, medical errors)					
I have the skills necessary to manage stress and prevent burnout					
I have the skills necessary to cope with setbacks and failures					
Difficult clinical events affect my well being					
I feel comfortable talking about stress and burnout with my peers					
I feel comfortable talking about medical errors I have been involved in, with my peers					
I think resilience training (learning how to adapt well to challenges) would be helpful					
I have had sufficient resilience training (learning how to adapt well to challenges)					

**We are designing workshops to teach students resilience skills, mark the 3 most important topics to you (areas that you need more training in)? (Mark ONLY 3)**

_Setting realistic goals_Managing expectations_Coping with difficult patient interactions

_Delivering bad news_Coping with medical errors _Coping with difficult team interactions (i.e. student, resident, attending)

_Dealing with disappointment/setbacks_Dealing with loss_Feeling gratitude

_Finding meaning in your daily work_Other: __________________

**When do you think this resiliency education would be most beneficial? (circle 1)**

Before 3^rd^ yearDuring 3^rd^ yearDuring 4^th^ year

**Suggestions: (not required to respond)**
